# Protective Impacts of *Chlorella vulgaris* on Cisplatin-Induced Toxicity in Liver, Kidney, and Spleen of Rats: Role of Oxidative Stress, Inflammation, and Nrf2 Modulation

**DOI:** 10.3390/life15060934

**Published:** 2025-06-10

**Authors:** Layla A. Almutairi, Ebtehal G. Abdelghaffar, Hany A. Hafney, Hala M. Ebaid, Sahar A. Alkhodair, Aly A. M. Shaalan, Heba N. Gad EL-Hak

**Affiliations:** 1 Department of Biology, College of Science, Princess Nourah bint Abdulrahman University, Riyadh 13412, Saudi Arabia; 2 Zoology Department, Faculty of Science, Suez Canal University, Ismailia 41522, Egypthany_ahmed@science.suez.edu.eg (H.A.H.);; 3 Department of Biochemistry, Faculty of Science, King Abdulaziz University, Jeddah 21589, Saudi Arabia; 4 Department of Anatomy, Faculty of Medicine, Jazan University, Jazan 45142, Saudi Arabia; 5 Department of Histology and Cell Biology, Faculty of Medicine, Suez Canal University, Ismailia 41522, Egypt

**Keywords:** cisplatin, Chlorella vulgaris, oxidative stress, inflammation, Nrf2 signaling

## Abstract

Cisplatin is a widely utilized chemotherapy drug effective against various cancers, yet its use is often constrained by severe toxicity to healthy organs, including the liver, kidneys, and spleen. This study explored the protective role of *Chlorella vulgaris*, a microalga known for its antioxidant and anti-inflammatory properties, against cisplatin-induced organ damage. The research focused on modulating oxidative stress, inflammation, and the Nrf2 signaling pathway. The experimental design included four groups: a control group receiving saline, a cisplatin group administered 1.34 mg/kg weekly for three months, a *C. vulgaris* group receiving 150 mg/kg daily, and a combined cisplatin/*Chlorella vulgaris* group. Cisplatin treatment significantly elevated oxidative stress markers, such as lipid peroxidation and nitric oxide, while increasing pro-inflammatory cytokines (TNF-α, IL-12, IL-6) and reducing antioxidant capacity. Additionally, liver and kidney function markers were markedly impaired, and histopathological analysis revealed structural damage in the liver, kidneys, and spleen. Conversely, *C. vulgaris* supplementation mitigated these effects, restoring oxidative stress markers, cytokine levels, and organ function to near-normal values. Microscopic examination confirmed that *Chlorella vulgaris* effectively prevented cisplatin-induced structural damage. Notably, while cisplatin increased Nrf2 expression as an adaptive response to oxidative stress, *C. vulgaris* attenuated this effect, reflecting its potent antioxidant capabilities.

## 1. Introduction

Chemotherapy is a widely used treatment for cancer, either as a standalone therapy or in combination with other modalities such as surgery and radiotherapy [1]. It employs a broad range of cytotoxic agents that target and damage cancerous tissues [2]. Cisplatin has remained one of the most commonly used chemotherapy drugs due to its broad-spectrum efficacy against various cancers [3]. Cisplatin effectively treats solid tumors, hematological malignancies, bladder, head and neck, esophageal, gastric, pulmonary, testicular, and ovarian cancers, lymphoma, and osteosarcoma [4]. Its mechanism of action involves the formation of covalent adducts between platinum and purine bases in DNA, which induces G2 cell cycle arrest and triggers apoptosis [5]. Despite its established efficacy as an antineoplastic agent, cisplatin’s therapeutic application faces significant limitations due to its toxicity profile and pharmacokinetic properties. The drug exhibits a short half-life (<24 h) in humans, with pharmacokinetic studies revealing rapid tissue distribution, particularly to the kidneys, liver, intestines, and testes. Over 90% of circulating platinum becomes bound to plasma proteins, substantially affecting its bioavailability [6]. Elimination kinetics demonstrate minimal urinary excretion during the initial 6 h post-administration, with similarly negligible biliary and intestinal clearance pathways. This distribution and clearance profile contributes to cisplatin’s characteristic toxicity pattern in various organ systems, necessitating careful clinical monitoring and consideration of protective interventions [7,8].

Despite its proven therapeutic effectiveness, cisplatin is associated with significant toxicity to healthy tissues, including the liver, kidneys, spleen, testes, nervous system, and hematological system, even when administered at clinically effective doses [9]. Within days of starting treatment, approximately one-third of patients receiving cisplatin experience a decrease in glomerular filtration rate and a range of other side effects [10]. Despite these adverse effects, cisplatin remains one of the most widely used chemotherapy agents, leading to ongoing debate about whether its therapeutic benefits outweigh the associated risks. Developing safer and more effective antineoplastic agents remains a major challenge in cancer treatment. As a result, there is growing interest in combining traditional cancer therapies like cisplatin with natural remedies to enhance their therapeutic efficacy while minimizing systemic toxicity. This approach often involves reducing drug doses and decreasing side effects [11].

One promising natural remedy is *Chlorella vulgaris*, a unicellular freshwater microalga rich in essential nutrients such as proteins, carbohydrates, vitamins, antioxidants, fatty acids, and trace elements [12]. Key antioxidants found in *C. vulgaris* include beta-carotene, chlorophyll, ascorbic acid, tocopherol, phenolic compounds, astaxanthin, flavonoids, and vital minerals like zinc, copper, and magnesium. These compounds play a crucial role in supporting the activity of antioxidant metalloenzymes [13]. Due to its nutrient profile and health benefits, *C. vulgaris* is commonly used as a functional food or dietary supplement. Its positive effects on human health including improved immune function, detoxification, and overall vitality have been well documented [14].

While extensive research has documented cisplatin-induced organotoxicity and the antioxidant properties of *C. vulgaris*, the current study addresses critical gaps by investigating chronic exposure scenarios and systemic protective mechanisms. Previous investigations have predominantly focused on acute toxicity models, leaving the cumulative pathophysiological sequelae of prolonged cisplatin administration inadequately characterized. Our three-month experimental paradigm uniquely models the sustained chemotherapy regimens encountered clinically, enabling assessment of *C. vulgaris* prophylactic potential against progressive multi-organ damage. Furthermore, while existing literature emphasizes single-organ analyses, we implemented a comparative interorgan approach evaluating hepatic, renal, and splenic responses simultaneously. Most innovatively, this study elucidates the previously unreported modulatory effects of *C. vulgaris* on the Nrf2-mediated oxidative stress response pathway during cisplatin challenge, proposing a novel mechanism whereby algal supplementation attenuates the initial redox imbalance rather than merely enhancing compensatory antioxidant defenses. We hypothesized that chronic dietary *C. vulgaris* supplementation would mitigate cisplatin-induced multi-organ dysfunction through coordinated suppression of oxidative stress biomarkers, pro-inflammatory cytokines, and dysregulated Nrf2 signaling across hepatic, renal, and splenic tissues. Our research investigated the effects of cisplatin using an experimental animal model to enhance our understanding of its systemic impact. By examining the spleen, kidney, and liver responses, we aimed to detect subtle indicators of toxicity that might not be immediately evident in clinical settings. This comprehensive approach provides valuable insights that could lead to improved strategies for managing treatment-related side effects and enhancing patient care outcomes. This study aimed to investigate the protective potential of *C. vulgaris* against cisplatin-induced organ damage, specifically focusing on its ability to mitigate oxidative stress inflammation and modulate the Nrf2 signaling pathway, a key regulator of the body’s antioxidant defense mechanisms. The results of this study may elucidate the role of *C. vulgaris* as a supplemental treatment to lessen the harmful effects of cisplatin while augmenting its anticancer efficacy.

## 2. Materials and Methods

### 2.1. Experimental Animals

Before the experiment commenced, 20 male Sprague-Dawley rats weighing 200 and 250 g were acclimatized to the laboratory setting for 7 days. The rats were kept in polyethylene cages featuring well-ventilated lids, maintained at 25 ± 5 °C, and exposed to a standard 12-h light/dark cycle. They received commercial food pellets and had unlimited access to tap water. The investigation complied with the ethical standards for using and caring for laboratory animals as specified by the European Community guidelines. The Faculty of Science Ethical Committee at Suez Canal University in Ismailia, Egypt, approved (approval number: REC66/2020). Throughout the acclimatization phase, the body weight of each rat was measured with a precise scale.

### 2.2. Chemicals

#### 2.2.1. Cisplatin

Benta Pharma Industries, Lebanon, supplied the cisplatin drug (cis-diamminedichloroplatinum II) used in this study. Mylan markets it as a concentrated solution for infusion or injection in vials.

#### 2.2.2. Chlorella

Chlorella tablets (*C. vulgaris*) were obtained from NOW FOODS, Bloomingdale, IL, USA. Each tablet contains 500 mg of organic chlorella algae. The tablets were dissolved in distilled water and administered to the rats via oral gavage at a daily dose of 150 mg/kg, as selected according to Zainul Azlan et al. [15]. The total phenolic including flavonoid and flavonoid contents only were determined following the methods described by Yadavalli et al. [16]. The extract was analyzed using a Waters 2690 Alliance HPLC system as detailed in [17]. The HPLC analysis employed a C18 Inertsil ODS column (4.6 × 250 mm, 5 µm particle size). The study used a stock solution containing 10 different standards dissolved in methanol: gallic acid, catechin, chlorogenic acid, rutin, ellagic acid, hesperidin, caffeic acid, apigenin, kaempferol, and quercetin. The selection of these 10 phenolic compounds was based on their documented prevalence in *C. vulgaris* extracts, their well-established antioxidant and anti-inflammatory activities, and the availability of reliable analytical standards [18]. These compounds have been frequently reported in previous phytochemical studies of *C. vulgaris* and are relevant to the mechanisms underlying cisplatin-induced toxicity.

### 2.3. Experimental Groups and Design of Work

Following an adaptation period of one week, the animals were separated into four groups, each consisting of six rats. The animals received a saline injection intraperitoneally as part of the control group. The cisplatin group received intraperitoneal injections of 1.34 mg/kg of cisplatin once weekly for three months. This quantity of cisplatin was similar to the human treatment regimen, and the dosage was 20 mg after conversion. We have decided to use a dosage of cisplatin that is 1/10 of the LD50. Following the rules established by the Organization for Economic Co-operation and Development (OECD) for acute oral toxicity testing of chemicals, the determination was carried out by means of an up-and-down technique [19]. Chlorella group: The animals received 150 mg/kg of chlorella orally for 3 months. Chlorella/Cisplatin group: The animals received 150 mg/kg chlorella orally for 3 months and were injected intraperitoneally with 1.34 mg/kg cisplatin once weekly for 3 months.

#### 2.3.1. Hematological Sampling

Blood samples were obtained from the medial retro-orbital venous plexus of rats using Micro Hematocrit Capillaries while under anesthesia with (100 mg/Bwt) thiopental sodium [20] for clinical biochemistry assays [21]. After blood sampling, the animals were dissected to acquire organs for histological, immunohistochemical, and ultrastructure examinations.

#### 2.3.2. Body Weight and Absolute Organ Weight

The body weight of each rat was assessed using a sensitive balance during the acclimatization period, once before the commencement of dosing, once weekly during the dosing period, and once on the day of sacrifice. The weights of the absolute organs, specifically the liver, kidneys, and spleen, were measured using a sensitive balance.

#### 2.3.3. Clinical Biochemical Studies

To obtain serum aliquots, one blood sample was collected from each control and treated rats into a simple gel tube and left at room temperature for an hour. The serum was pipetted off after the tubes had been centrifuged at 5000 rpm for 10 min, and they were then stored at −20 °C until they were used. For all the clinical biochemical studies, control rats were included in each assay batch to establish baseline values. All readings were normalized to total protein concentration to account for any variations in sample volume. Six rats were included in each treatment group.

##### Liver Function Tests

Blood bilirubin concentration was conducted using a commercially available kit (Diamond, Diagnostics company, Giza, Egypt, REF: 265 mL) based on the quantitative determination method for bilirubin [22], direct and indirect bilirubin according to [23]. Blood Alanine aminotransferase (ALT) concentration using UV kinetic method [24], blood Aspartate aminotransferase (AST) concentration using UV kinetic method [25], total protein concentration was performed using the Biuret colorimetric endpoint method [26], blood Albumin concentration using BCG colorimetric method [27] and the total globulin concentration [28] were measured using as calculated value (Total Protein-Albumin).

##### Kidney Function Tests

The determination of Blood urea concentration using Enzymatic UV test (Urease-GLDH method) [29], Serum Uric Acid using Enzymatic colorimetric test (Uricase method) [30], and creatinine concentration using Colorimetric method [31] were measured using a commercial Roche Diagnostics kit.

##### Lipid Profile Tests

The measurements of serum cholesterol using CHOD-PAP enzymatic colorimetric method [32], Triglycerides using GPO-PAP enzymatic colorimetric method [33], and LDL using Direct Method Selective detergent method [34].

##### Ions of Blood

Serum sodium (Na), potassium (K), and calcium (Ca++) were estimated using (ST-200 Plus) electrolyte analyzer (Code/Reagent pack: ST-200 Pro/Plus/CL) provided by SENSA CORE Company (Hyderabad, India) for healthcare and diagnostic products [35]. Serum total calcium is estimated by a commercial kit SPINREACT (MDBSIS09-I 25/04/17 SPINREACT, S.A./S.A.U. Ctra. Santa Coloma, 7 E-17176 Sant Esteve De Bas (GI), Spain) [36].

##### Inflammatory Cytokine, Oxidative Biomarker and Total Antioxidant Parameters

TNF-α was determined in the blood serum using an ELISA kit (products of Cusa Biotechnology LLC, Houston, TX, USA, CAT. NO. TA CSB-E11987r) according to the method of Perez-Gracia et al. [37]. MDA was determined according to Jakovljevic et al. [38]. TAC provides an overall measure of antioxidant status of both enzymatic and non-enzymatic antioxidants, reflecting the cumulative effect of all antioxidants present. TAC was measured using Cayman Chemical kits CAT. Antioxidant Assay Kits: 709001. Levels of NO in the serum were determined using a commercial kit (Biodignostic, Giza, Egypt, CAT. NO. TA 2533) according to the method of Rubio et al. [39]. ELISA Kit Catalog Number CSB-E07364r for the quantitative determination of rat interleukin 12 (IL-12) concentrations, IL6 ELISA kit Cat. No. KT19418 for the quantitative determination of rat IL6 in serum according to Sakai et al. [40].

#### 2.3.4. Histological and Immunohistochemical Preparation

After sacrifice, each animal’s liver, kidney, and spleen were cleaned, rinsed with saline, and preserved in 10% neutral buffered formalin. After overnight processing, they were standard histologically. We utilized a microtome to cut 5-micron slices and mounted them on glass slides for histology and charged slides for immunohistochemistry.

##### Histological Preparation

The tissue sections were stained with Hematoxylin and Eosin (H and E) [41]. Finally, the slides were cleared with xylene and mounted using DPX. Blind pathologists grade the histopathological scoring of the liver, kidney, and spleen according to the percentage of the affected area to normal = 0, mild to less than 25%, moderate from 25% to 50%, and severe to more than 50% [6].

##### Immunohistochemical Preparation

Immunohistochemical detection of nuclear factor-erythroid-related factor 2 (Nrf2) used a monoclonal anti-Nrf2 antibody (Cat. No. ab137550 Abcam, Cambridge, UK) diluted 1:1000–1:2500 overnight at 4 °C by routine immunohistochemical methods as described in Nunes de Melo et al. [40]. Slides were examined and photographed. Immunohistochemical expression was quantified using the Fiji/ImageJ program, which measured the proportion of immunopositive cells per square millimeter. Nrf2 immunoreactivity was assessed at 400× magnification by analyzing 10 non-overlapping areas per slice.

##### Electron Microscopy Analysis

Small liver, kidney, and spleen samples were excised and subsequently sectioned in the presence of 2% glutaraldehyde using a dissecting microscope. A 2% glutaraldehyde fixative solution in 0.1 M Na-cacodylate buffer was applied to the specimens for a duration of 24 h. The specimens were washed in phosphate buffer, post-fixed with osmium tetroxide, and infiltrated with resin. They were dehydrated in ethyl alcohol, sectioned into slices using an ultramicrotome, stained with toluidine blue, and analyzed under a light microscope. Representative fields were analyzed using a transmission electron microscope at the Electron Microscope Unit [42]. The mitochondria, nuclei, rough endoplasmic reticulum, and smooth endoplasmic reticulum of the liver, kidney, and spleen were assessed.

### 2.4. Statistical Analysis of Data

The data were systematically organized and graphically presented utilizing Microsoft Excel XP. Statistical analysis involved comparing means using a One-way analysis of variance (ANOVA), followed by post-hoc Duncan’s test. Differences were assessed using SPSS version 11.0 for Windows. A *p*-value below 0.05 was deemed statistically significant, with all results reported as the mean ± standard error (SE) to provide an estimate of the precision of the sample mean.

## 3. Results

### 3.1. Total Phenolic and Flavonoid Compounds in C. vulgaris Supplement

The aqueous extract of *Chlorella C. vulgaris* was analyzed, revealing the presence of key phytochemicals, including phenolic compounds and flavonoids. The aqueous extract contained 70.56 mg of total phenolic compounds including flavonoids and 55.36 mg of flavonoids especially per gram of dry cell weight. The chromatographic analysis identified 10 phenolic compounds in the extract: gallic acid, catechin, chlorogenic acid, rutin, ellagic acid, hesperidin, caffeic acid, apigenin, kaempferol, and quercetin (Table 1 and Supplementary Figure S1).

### 3.2. Effect of Cisplatin and Chlorella Supplement on Clinical Observations, Body Weight, and Organ Weights (Liver, Kidney, and Spleen)

**Figure 1:**
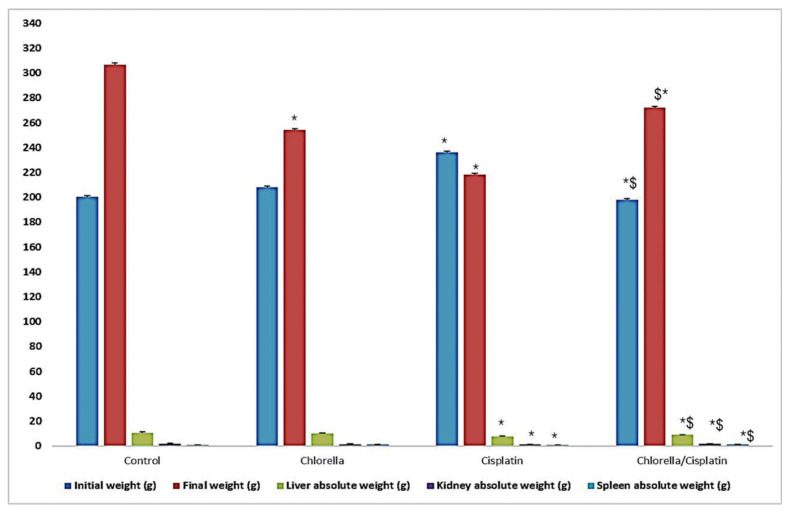
Effect of Cisplatin and Chlorella supplement treatment on body, liver, kidney and spleen weights. Data were expressed as means ± SEM, *n* = 6. Data were statically analyzed using One-way ANOVA followed by Duncan’s multiple comparisons test. * Indicates *p* < 0.05 compared to the control group. $ indicates *p* < 0.05 compared to the cisplatin group.

Clinical signs of cisplatin toxicity were observed in the rat’s administered cisplatin, including aggressive behavior, fatigue, ataxia, fur loss, and polyuria. These signs were absent in control and chlorella-treated groups. The effect of cisplatin and chlorella supplementation on rat body weight and the weight of major organs (liver, kidney, and spleen) is presented in Figure 1. Cisplatin treatment caused a considerable decrease in the liver, kidney, and spleen weights compared to the control group. However, pre-treatment with chlorella before cisplatin administration significantly (*p* ≤ 0.05) increased the body weight and liver, kidney, and spleen weights compared to the cisplatin-only group.

### 3.3. Effect of Cisplatin and Chlorella Supplement on Biochemical Parameters

#### 3.3.1. Hepatocellular Injury Markers

**Figure 2:**
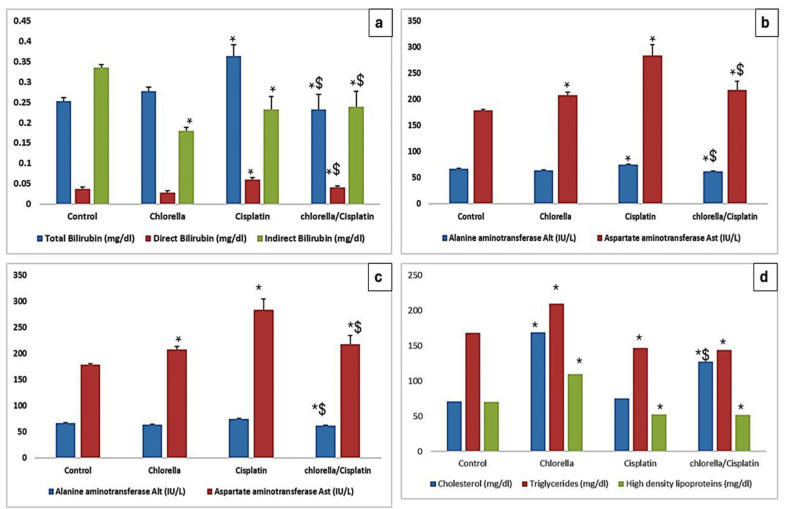
Effect of Cisplatin and Chlorella supplement treatment on (**a**–**c**) the measured marker of the hepatocellular injury and (**d**) some measured lipid profile (cholesterol, triglycerides, and low-density lipoproteins (LDL)). Data were expressed as means ± SEM, *n* = 6. Data were statically analyzed using One-way ANOVA followed by Duncan’s multiple comparisons test. * Indicates *p* < 0.05 compared to the control group. $ indicates *p* < 0.05 compared to the cisplatin group.

The effects of Cisplatin and Chlorella supplementation on liver function parameters (total bilirubin, direct bilirubin, indirect bilirubin, ALT, AST, albumin, globulin, and total protein) are shown in Figure 2. In the cisplatin group, the indicators of liver function were markedly increased. In contrast, the chlorella/cisplatin group showed a significant improvement, with values approaching those of the control group.

#### 3.3.2. Lipid Profile

The unique effects of Cisplatin and Chlorella supplementation on rats’ lipid profiles are detailed in Figure 2d. There was a substantial increase (*p* < 0.05) in the levels of serum cholesterol, triglycerides, and low-density lipoprotein (LDL) in the group that was administered cisplatin in comparison to the group that served as the control. On the other hand, the chlorella/cisplatin group showed a considerable reversal of these anomalies in the lipid profile, with values coming closer to those reported in the control group.

#### 3.3.3. Kidney Function

**Figure 3:**
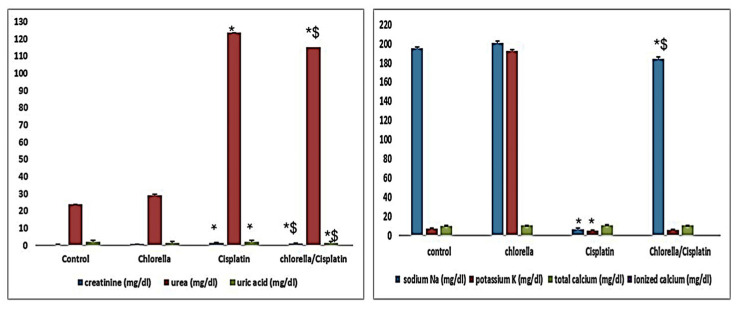
Effect of Cisplatin and *C. vulgaris* supplement treatment on Kidney Functions (creatinine, urea and uric acid) and Blood Ions (sodium, potassium and calcium). Data were expressed as means ± SEM, *n* = 6. Data were statically analyzed using One-way ANOVA followed by Duncan’s multiple comparisons test. * Indicates *p* < 0.05 compared to the control group. $ indicates *p* < 0.05 compared to the cisplatin group.

Figure 3 summarizes the impact of Cisplatin and Chlorella supplementation on kidney function parameters such as uric acid, urea, and creatinine. There was a statistically significant difference (*p* ≤ 0.05) in serum urea and creatinine levels between the cisplatin and control groups. There was a little but not statistically significant rise in serum uric acid levels in the cisplatin group. On the other hand, the chlorella/cisplatin group demonstrated a noteworthy decrease in blood levels of uric acid and creatinine compared to the cisplatin group. Even though there was a reduction in serum urea in the group that received chlorella and cisplatin, this change did not satisfy the criteria for statistical significance.

#### 3.3.4. Blood Ions

Figure 3 also illustrates the effects of supplementation with chlorella and cisplatin on the levels of blood ions (Na, K, Ca, and ionized calcium). Comparing the blood levels of sodium and potassium in the cisplatin group to those in the control group revealed a significant difference. On the other hand, the chlorella/cisplatin group had a rise in sodium and potassium levels comparable to those of the control group. The total and ionized calcium levels of the control and treatment groups did not vary significantly from one another in any meaningful way.

### 3.4. Effect of Cisplatin and Chlorella Supplement on an Oxidative Biomarker, Total Antioxidant Parameters, and Inflammatory Cytokine in the Serum of Rats

**Figure 4:**
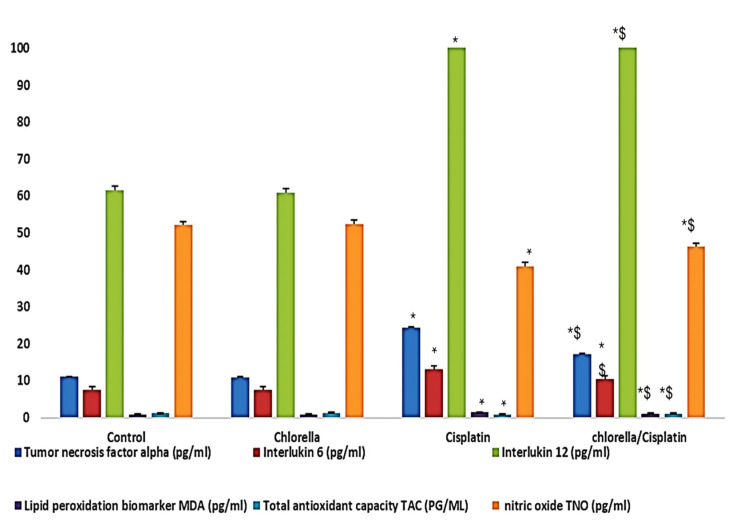
Effect of Cisplatin and Chlorella supplement treatment on serum total antioxidant capacity (TAC), lipid peroxidation biomarker malonaldehyde (MDA), Total nitric oxide (NO), Tumor necrosis factor (alpha) (TNF-α), Interlukin-6 (IL-6) and Interlukin-12 (IL-12). Data were expressed as means ± SEM, *n* = 6. Data were statically analyzed using One-way ANOVA followed by Duncan’s multiple comparisons test. * Indicates *p* < 0.05 compared to the control group. $ indicates *p* < 0.05 compared to the cisplatin group.

Figure 4 summarizes the blood levels of TAC, MDA, NO, TNF-α, IL-6, and IL-12 for both the control and treatment groups. Compared to the control group, the mean levels of MDA, NO, TNF-α, IL-6, and IL-12 of the cisplatin group were considerably greater than those of the control group. As an additional point of interest, the mean TAC level in the cisplatin group was significantly lower than in the control group. In comparison to the cisplatin group, the use of chlorella as a pre-treatment prior to the administration of cisplatin resulted in a substantial reduction (*p* < 0.05) in the levels of MDA, NO, TNF-α, IL-6, and IL-12. Nevertheless, compared to the group that only received cisplatin, the group that received chlorella and cisplatin demonstrated a significant decrease in TAC levels (*p* < 0.05).

### 3.5. Histological Examination

#### 3.5.1. Histological Examination of Liver

**Figure 5:**
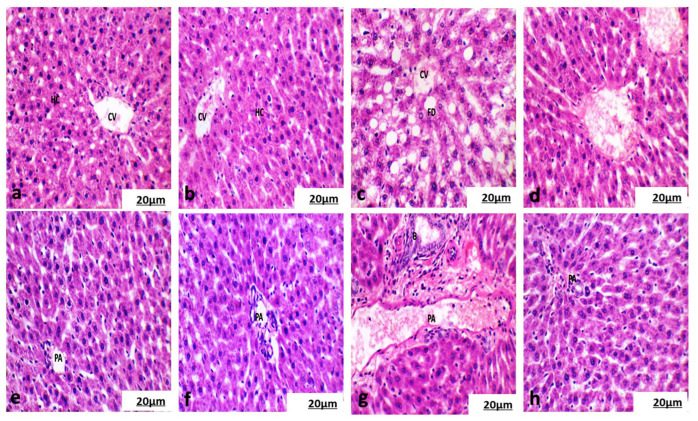
(**a**) Photomicrographs of the liver showed a normal structure with normal hepatocytes (HC) surrounding the central vein (CV) in the control rat. (**b**) Photomicrographs of the liver showed a normal structure with the normal portal area (PA) and bile duct in the treated rat with chlorella. (**c**) Photomicrographs of the liver showed the altered structure with fatty degenerated hepatocytes (FD) surrounding the central vein (CV) from rats treated with cisplatin. (**d**) Photomicrographs of the liver showed normal structure with normal hepatocytes surrounding the central vein to the treated group with cisplatin and chlorella. (**e**) Photomicrographs of the liver showed normal hepatocytes near the portal area (PA) from control rats. (**f**) Photomicrographs of the liver showed normal structure with normal hepatocytes surrounding the portal area (PA) from the chlorella-treated rats. (**g**) Photomicrographs of the liver showed infiltration of the hepatocytes surrounding the portal area (PA) with proliferation and hypertrophy of the bile duct (B) from rats treated with cisplatin. (**h**) Photomicrographs of the liver showed normal structure with normal hepatocytes surrounding the portal area (PA) from the chlorella/cisplatin group (H and E × 400).

Figure 5a–d show no histopathological alterations in the liver tissue of the control and chlorella groups. The liver of the cisplatin-treated group demonstrated hydropic and fatty degeneration of hepatocytes adjacent to the central vein, along with invasion of inflammatory cells in the periportal triad region. (Figure 5e,f). Examination of liver tissue from the chlorella/cisplatin group revealed that the hepatic lobules remained intact and appeared structurally normal, similar to the control group (Figure 5,h).

#### 3.5.2. Histological Examination of the Cortical Region of Kidney

**Figure 6:**
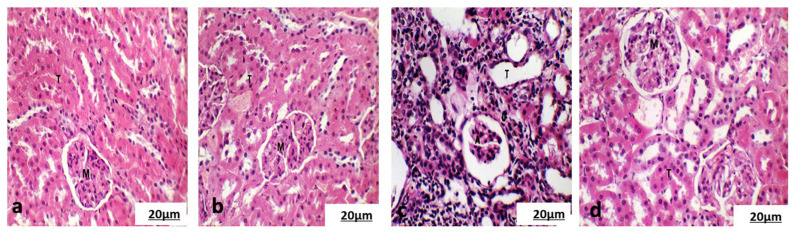
(**a**) Photomicrographs of the kidney showed a normal Malpighian corpuscles structure (M) with a normal glomerulus surrounded with proximal convoluted tubules (T) from the control group. (**b**) Photomicrographs of the kidney showed a normal Malpighian corpuscles structure (M) with a normal glomerulus surrounded with proximal convoluted tubules (T) from the control group. (**c**) Photomicrographs of the kidney showed atrophic glomerulus (A) surrounded with dilated proximal and distal convoluted tubules and infiltration of inflammatory cells between tubules (IF) from the cisplatin group. (**d**) Photomicrographs of the kidney showed a normal Malpighian corpuscles structure (M) with a normal glomerulus surrounded with proximal convoluted tubules (T) from the chlorella/cisplatin group. (H and E × 400).

Examination of the kidneys from the control and chlorella groups (Figure 6a,b) showed regular renal corpuscles (Malpighian corpuscles) and well-preserved renal parenchyma. The kidneys from the cisplatin-treated group showed significant histological changes, including foamy cytoplasm, hydropic degeneration of some tubules, invasion of inflammatory cells between the tubules, and dilatation of the convoluted tubules. The glomerular capillary layer also exhibited atrophy and fusion (Figure 6c). However, the renal tissue in the chlorella/cisplatin group appeared normal, with no significant alterations in the kidney architecture (Figure 6d).

#### 3.5.3. Histological Examination of Spleen

**Figure 7:**
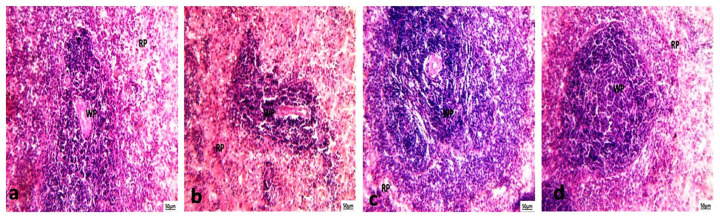
(**a**) Photomicrographs of the spleen of the control rat showed normal white pulp (WP) and red pulp (RP). (**b**) Photomicrographs of the spleen of a treated rat with chlorella showed normal white pulp (WP) and red pulp (RP). (**c**) Photomicrographs of the spleen of a treated rat with cisplatin showed marginal zone hyperplasia of the white pulp (WP) and normal hematogenous red pulp (RP). (H and E × 200). (**d**) Photomicrographs of the spleen of treated rats with chlorella and cisplatin showed normal white pulp (WP) and red pulp (RP). (H and E × 200).

Histopathological analysis of spleen sections from the control and chlorella groups demonstrated intact splenic architecture, characterized by well-defined lymphoid white pulp featuring lymphoid follicles and periarteriolar lymphoid sheaths surrounding the central artery alongside normal red pulp (Figure 7a,b). In the cisplatin-treated group, however, there was evidence of marginal zone hyperplasia in the white pulp, while the red pulp remained normal (Figure 7c). In the chlorella/cisplatin group, the spleen showed a normal white and red pulp distribution, similar to the control group (Figure 7d).

### 3.6. Immunohistochemical Expression Nrf2 of Liver and Kidney

**Figure 8:**
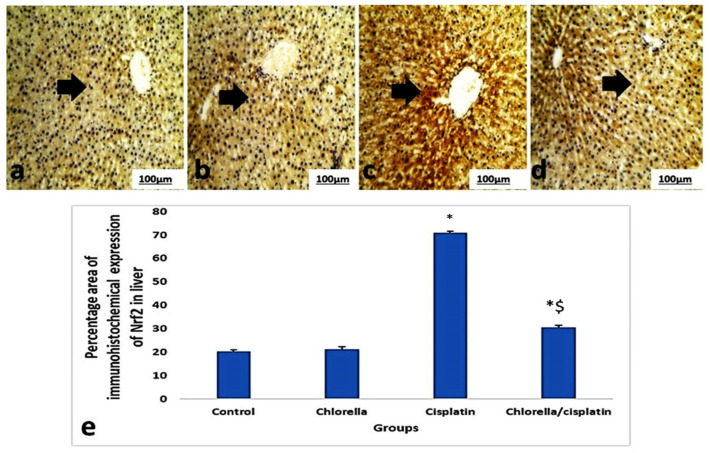
Immunoexpressing nuclear erythroid factor 2 (Nrf2) in the sections of the liver and kidney of rats belonging to the control group and treated groups. (**a**,**b**) Liver sections of control rats and rats treated with chlorella showed mild brown immunoexpressing of Nrf2 in the hepatocytes (arrow). (**c**) Liver sections of rats treated with cisplatin showed severe brown immunoexpressing of Nrf2 in the hepatocytes (arrow). (**d**) Liver sections of the group pretreated with chlorella before injection with cisplatin showed mild brown immunoexpressing of Nrf2 in the hepatocytes (arrow). (200×). (**e**) Histogram of the mean percentage areas of Nrf2 protein expression in the hepatocytes of different groups. * Indicates *p* < 0.05 compared to the control group. $ indicates *p* < 0.05 compared to the cisplatin group.

**Figure 9:**
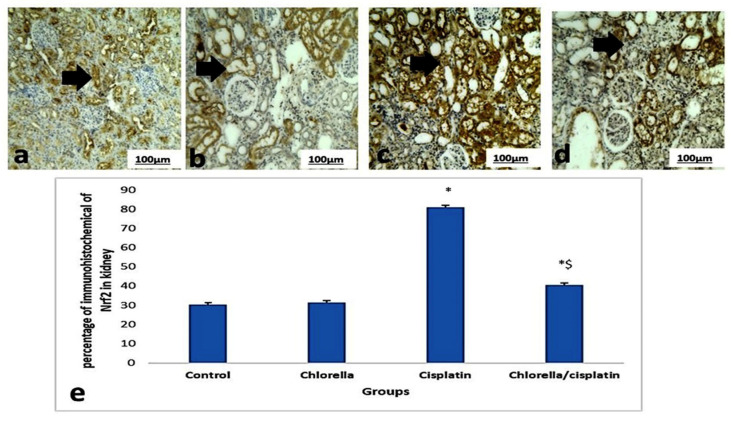
Immunoexpressing of nuclear erythroid factor 2 (Nrf2) in the sections of the kidney of rats belonging to the control group and treated groups. (**a**,**b**) The kidney section of control rats and rats treated with chlorella showed mild brown immunoexpressing of Nrf2 in the kidney (arrow). (**c**) The kidney section of rats treated with cisplatin showed severe brown immunoexpressing of Nrf2 in the kidney (arrow). (**d**) Kidney sections of the group pretreated with chlorella before injection with cisplatin showed mild brown immunoexpressing of Nrf2 in the kidney (arrow). (200×). (**e**) Histogram of the mean percentage areas of Nrf2 protein expression in the kidneys of different groups. * Indicates *p* < 0.05 compared to the control group. $ indicates *p* < 0.05 compared to the cisplatin group.

Figure 8 and Figure 9 illustrated the brown staining in hepatocytes and the portal areas of the liver and kidney, reflecting the immunohistochemical assessment of the Nrf2 pathway in control and treated rats. Only minimal Nrf2 expressions were observed in the liver and kidney sections in the control and chlorella groups. However, cisplatin treatment significantly increased Nrf2 expression (*p* ≤ 0.05), as implied by intense brown cytoplasmic staining in hepatocytes and renal cells. In contrast, when administered before cisplatin, the chlorella pre-treatment group showed only mild Nrf2 expression in both hepatocytes and kidney cells, compared to the cisplatin-only group.

### 3.7. Ultrastructure Examination

#### 3.7.1. Ultrastructure Changes in Liver

**Figure 10:**
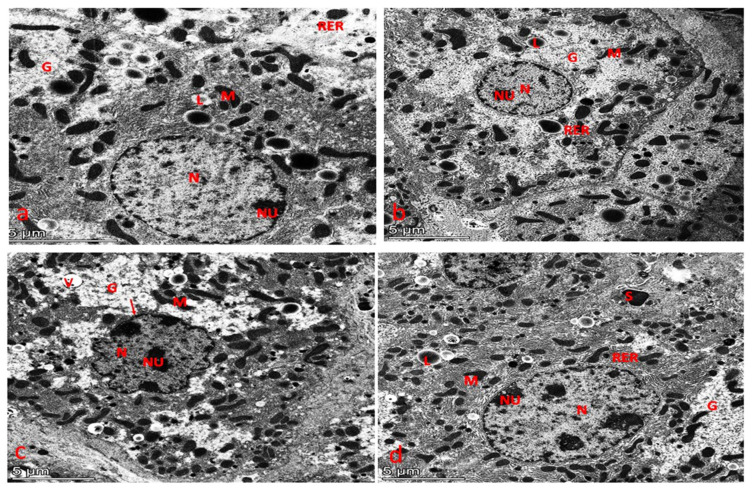
(**a**) An electron micrograph of the liver from the control group showed ideal hepatocytes with a normally round nucleus (N) and nucleolus with evenly distributed chromatin, sometimes slightly condensed along the nuclear membrane in hepatocytes; the nucleus is easily distinguished. There are numerous round, oval, elongated rod-like mitochondria (M) with membranous cristae and electron-dense matrix. The normal rough endoplasmic reticulum (RER) and the smooth endoplasmic reticulum occurred in glycogen-rich areas (G). (**b**) An electron micrograph of the liver from the chlorella group showed exhibited ideal hepatocytes with normally round nucleus (N) and nucleolus (Nu) with evenly distributed chromatin, sometimes slightly condensed along the nuclear membrane in hepatocytes; the nucleus is easily distinguished. There are numerous round, oval, elongated rod-like mitochondria (M) with membranous cristae and electron-dense matrix and lipid droplets (L). The normal rough endoplasmic reticulum (RER) and the smooth endoplasmic reticulum occurred in glycogen-rich areas (G). (**c**) An electron micrograph of the liver from the cisplatin group showed ultrastructure alteration, including irregularity to the contour of the nucleus (N) with dense nucleolus (NU). Shrunken nuclei (arrow) with condensed chromatin in the hepatocyte were seen. The cytoplasm was vacuolated (V) and dissolute with increased glycogen granules (G). Numerous round, oval, elongated rod-like mitochondria (M), and lipid droplets are present. (**d**) An electron micrograph of the liver from the chlorella/cisplatin group showed ideal hepatocytes and sinusoid (S). The hepatocytes have a normally round nucleus (N) and nucleolus (Nu) with evenly distributed chromatin, sometimes slightly condensed along the nuclear membrane in hepatocytes; the nucleus is easily distinguished. Numerous round, oval, elongated rod-like mitochondria (M) matrix, and lipid droplets (L) are presented. The normal rough endoplasmic reticulum (RER) and the smooth endoplasmic reticulum occurred in glycogen-rich areas (G).

Ultrastructural examination of liver sections from the control and chlorella groups revealed healthy hepatocytes with round nuclei and nucleoli, containing evenly distributed chromatin, which was occasionally slightly condensed along the nuclear membrane. The nuclei were well-defined, and numerous mitochondria with round, oval, or elongated rod-like shapes and well-developed cristae and electron-dense matrices were observed. The rough and smooth endoplasmic reticula were present, particularly in glycogen-rich areas (Figure 10a,b).

In contrast, the liver sections from the cisplatin-treated group (Figure 10c) exhibited several ultrastructural abnormalities. The nuclei of hepatocytes appeared irregular in shape, with shrunken nuclei and condensed chromatin. The cytoplasm was vacuolated, and there was an increase in glycogen granules. Additionally, the hepatocytes contained numerous mitochondria of various shapes along with lipid droplets.

In the liver sections from the protective and therapeutic groups (Figure 10d), the hepatocytes displayed a near-normal cellular structure, with most organelles appearing healthier compared to those in the cisplatin group. The hepatocytes had well-defined round nuclei and nucleoli, characterized by uniformly distributed chromatin, which was occasionally marginally compressed at the nuclear membrane. Mitochondria of various shapes, lipid droplets, and normal rough and smooth endoplasmic reticula in glycogen-rich areas were also present, resembling the structure observed in the control and chlorella groups.

#### 3.7.2. Ultrastructure Changes in the Kidney

**Figure 11:**
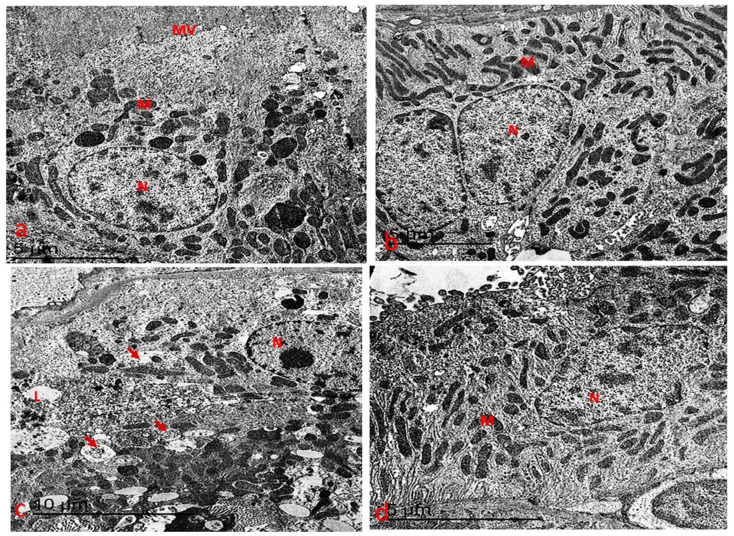
(**a**) An electron micrograph of the kidney from the control group showed convoluted tubules with an active nucleus (N) with nucleoli. Elongated mitochondria (M) on a highly developed basal membrane and a microvillus (MV) along the luminal border. (**b**) An electron micrograph of the kidney from the chlorella group showed convoluted tubules with an active nucleus (N) with nucleoli. Elongated mitochondria (M) on a highly developed basal membrane were observed. (**c**) An electron micrograph of the kidney from the cisplatin group showed convoluted tubules with a condensed chromatin nucleus (N), degenerated mitochondria and vacuolated cytoplasm (arrow). (**d**) An electron micrograph of the kidney from the chlorella/cisplatin group showed convoluted tubules with an active nucleus (N) with mildly irregular contour with nucleoli. Elongated mitochondria (M) on a highly developed basal membrane were observed.

Ultrastructural analysis of the cortical kidney sections from the control and chlorella groups revealed typical tubular structures (Figure 11a,b). In contrast, the cisplatin-treated group displayed significant ultrastructural changes in the tubular cells. These included cells with round nuclei, where the chromatin was evenly distributed but condensed along the nuclear membrane. Many of these nuclei appeared necrotic, with densely packed chromatin. Additionally, oval and elongated mitochondria with degenerated cristae (cristolysis) were observed. Cytoplasmic vacuolation was present in the cortical tubule cells, and there was a drop in the numeral of microvilli along the luminal border (Figure 11c).

In the chlorella/cisplatin group, ultrastructural examination returned to near-normal cortical cell architecture. The proximal and distal convoluted tubules exhibited typical cellular structures, with cells displaying irregularly contoured round nuclei and evenly distributed chromatin. While some mitochondrial cristae degeneration and mild cytoplasmic vacuolation were still present, the overall structure was notably improved (Figure 11d).

#### 3.7.3. Ultrastructure Changes of Spleen

**Figure 12:**
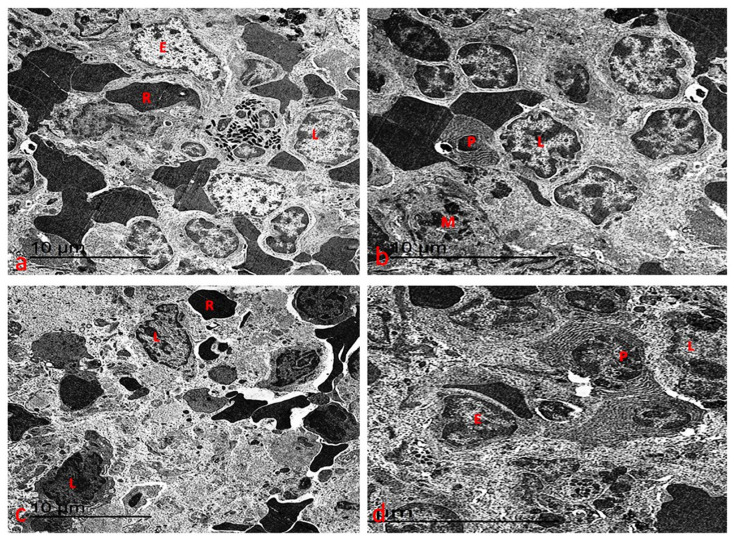
(**a**) An electron micrograph of the spleen from the control group showed normal ultrastructure of multiple lymphocytes of nuclei with chromatin that homogenously distributed (L). The red pulp is lined by endothelial cells (E) with red blood cells (R) between them. (**b**) An electron micrograph of the spleen from the chlorella group showed normal ultrastructure of multiple lymphocytes of nuclei with chromatin that homogenously distributed (L), macrophage (M), and plasma cells (P). The red pulp between its red blood cells. (**c**) An electron micrograph of the spleen from the control group showed lymphocytes with different degrees of marginal condensed chromatin in their nuclei. Most of the cytoplasm showed disintegrated organelles. (**d**) An electron micrograph of the spleen from the chlorella/cisplatin group showed normal ultrastructure of multiple lymphocytes of nuclei with chromatin that homogenously distributed (L) and plasma cells (P). The red pulp is lined with endothelial cells (E) between its red blood cells.

Ultrastructural analysis of the spleen in both control and chlorella-treated rats revealed a normal tissue architecture featuring a variety of lymphocytes, reticular cells, macrophages, neutrophils, and plasma cells (Figure 12a,b). In the cisplatin-treated group, however, the splenic tissue exhibited lymphocytes with varying degrees of marginal chromatin condensation in their nuclei. Most cells’ cytoplasm showed significant organelles disintegrating (Figure 12c). Notably, pre-treatment with chlorella before cisplatin administration largely restored the ultrastructure of the spleen to a pattern resembling that of the control rats, with most alterations reversed (Figure 12d).

## 4. Discussion

This study utilized a range of biochemical assessments, including dynamics of oxidants and antioxidants in the bloodstream, histopathological evaluations, and cellular analyses of liver, kidney, and spleen tissues, to investigate the potential protective effects of *C. vulgaris* supplementation against cisplatin-induced toxicity in healthy rats. This study explored the phenolic and flavonoid content of *C. vulgaris* supplements, revealing a predominance of beneficial phenolic compounds such as catechin, gallic acid, chlorogenic acid, hesperidin, caffeic acid, rutin, and ellagic acid. These compounds, identified in previous studies, are known for their potent antioxidant properties [43,44]. Comparable results were documented by Abdel-Karim et al. [45], who noted a broad range of phenolic and flavonoid compounds in the aqueous extracts of *C. vulgaris*.

Changes in body weight were used as an indicator of overall health status [46]. In the cisplatin-treated group, a significant reduction in the rate of weight gain was observed after 3 months, signaling disruption in normal metabolism [47]. This observation aligns with earlier studies that reported weight loss due to cisplatin’s metabolic interference [48]. In contrast, the group treated with both *C. vulgaris* and cisplatin showed a significant increase in body weight, which may reflect improved appetite or metabolic balance [49]. These findings corroborate those of Vijayavel et al. [50] and Hidaka et al. [51], who found that *C. vulgaris* supplementation improved body weight in experimental animals.

Organ weight measurement is a standard approach in toxicity studies, as changes in organ size can indicate toxic effects, often correlating with histopathological damage [52]. The cisplatin group showed significant changes in the weight of the liver, kidney, and spleen, consistent with signs of toxicity. These alterations in organ weight were predictive of histological damage, confirming the toxic effects of cisplatin. However, in the *C. vulgaris* and cisplatin group, the organ weights were significantly closer to control values, suggesting a protective effect of Chlorella, likely due to its antioxidant properties [45].

Liver function tests, including ALT, AST, albumin, and globulin markers, were used to assess potential hepatic toxicity [53]. Cisplatin administration significantly increased ALT and AST levels, indicators of hepatic damage. However, in rats pretreated with *C. vulgaris*, these enzyme levels were substantially lower, nearly equivalent to control levels, suggesting that Chlorella has substantial hepatoprotective effects. This finding aligns with the results of Omar et al. [54], who also observed elevated ALT and AST levels following cisplatin treatment. The cisplatin group also showed increased total protein, likely due to dehydration or stress-related protein accumulation [55], while serum albumin and globulin levels were elevated, potentially indicating autoimmune disease or cancer [56]. Rats pretreated with *C. vulgaris* showed restored normal values for total protein, albumin, and globulin, reinforcing the protective role of *C. vulgaris* in maintaining liver function.

Cisplatin-induced dyslipidemia is characterized by elevated cholesterol and triglyceride levels, which are commonly associated with oxidative stress, liver dysfunction, and other health complications. *C. vulgaris* supplementation significantly improved lipid profiles, lowering both total cholesterol and LDL cholesterol levels, likely due to its antioxidant and antihyperlipidemic properties, which may be linked to flavonoids. Similar lipid-modulating effects of *C. vulgaris* have been reported by Ebrahimi-Mameghani et al. [57].

Kidney function was assessed by measuring blood urea, creatinine, and uric acid levels [58]. In the cisplatin group, elevated levels of these markers indicated renal damage, likely resulting from increased protein catabolism or glomerular and tubular damage [18,59]. Administration of *C. vulgaris* prior to cisplatin treatment led to significant reductions in these markers, suggesting improved kidney function. Histological examination also revealed fewer signs of renal damage in the *C. vulgaris*-treated group, consistent with the nephroprotective effects of *C. vulgaris* observed in other studies [18,60,61].

Cisplatin-induced electrolyte disturbances, including reduced Na and K levels, were also noted in the present study. This may result from impaired tubular reabsorption and increased vascular resistance caused by cisplatin’s oxidative stress [62]. However, *C. vulgaris* treatment before cisplatin administration improved electrolyte reabsorption, likely due to its antioxidant properties, protecting against oxidative kidney damage.

Oxidative stress is a well-established mechanism of cisplatin toxicity, with reactive oxygen species contributing to cellular damage [63,64]. In the current study, cisplatin treatment increased MDA and reduced the TAC in the blood. However, *C. vulgaris* supplementation restored both MDA levels and TAC, suggesting its antioxidant potential. This aligns with prior reports by A El-Chaghaby et al. [65], who demonstrated that *C. vulgaris* contains polyphenols and flavonoids with antioxidant properties. *C. vulgaris* is known to contain a wide range of bioactive compounds, including antioxidants, vitamins, and minerals, which could interact with the assays in various ways. For example, the antioxidant properties of *C. vulgaris* components such as beta-carotene, chlorophyll, flavonoids, and phenolic compounds might alter the results of oxidative stress markers and cytokine assays. These compounds may interfere with assay reactions by scavenging free radicals, potentially leading to the reduction in observed oxidative stress levels and thus falsely suggesting a protective effect.

Cisplatin treatment also induced the discharge of pro-inflammatory cytokines, including TNF-**α**, IL-6, and IL-12, which play a role in organ damage. The increase in these cytokines was linked to oxidative stress and inflammation. However, *C. vulgaris* treatment before cisplatin administration dramatically decreased the levels of these pro-inflammatory markers, demonstrating its anti-inflammatory effects, which align with findings from Sibi and Rabina [66]. *C. vulgaris*’s anti-inflammatory and antioxidant properties likely contributed to the improvement in the cytokine profile observed in this study.

Histopathological analyses revealed cisplatin-induced damage to the liver, kidney, and spleen. Cisplatin causes hydropic degeneration, fatty infiltration, and inflammatory cell infiltration in the liver, classic signs of hepatotoxicity. *C. vulgaris* treatment protects against these changes, restoring normal liver histology. Similarly, cisplatin caused glomerular and tubular damage in the kidneys, which was mitigated by *C. vulgaris* treatment. The spleen also showed changes in the marginal zone following cisplatin administration, but these changes were less pronounced in the *C. vulgaris*-treated group.

Ultrastructural changes in the liver, kidney, and spleen cells were also observed. In the liver, cisplatin caused nuclear condensation and irregular cytoplasmic membranes, which were reversed mainly by *C. vulgaris* administration. Similarly, *C. vulgaris* treatment improved mitochondrial and tubular structure in the kidneys, reducing damage induced by cisplatin. In the spleen, *C. vulgaris* restored normal cellular morphology, counteracting the ultrastructural alterations caused by cisplatin.

Finally, *C. vulgaris* treatment modulated the Nrf2 pathway, a key oxidative stress response regulator [67]. In cisplatin-treated rats, Nrf2 immunoreactivity was increased, suggesting a cellular attempt to counteract oxidative damage. In contrast, rats pretreated with *C. vulgaris* showed reduced Nrf2 activation, indicating a protective effect against cisplatin-induced oxidative stress. While the results from this study indicate that *C. vulgaris* supplementation may have protective effects against cisplatin-induced toxicity, it is important to acknowledge the possibility that the complex ingredients of *C. vulgaris* might influence the assays used, potentially mimicking the protective effects observed.

Despite the promising findings of this study, several limitations should be acknowledged. First, molecular analyses, such as Western blotting or RT-PCR for tissue-specific inflammatory and oxidative stress markers, were not performed, which restricts our ability to confirm the mechanistic pathways underlying the observed protective effects of *C. vulgaris*. Second, the study duration was limited, and we did not assess long-term toxicity or cumulative effects, leaving the long-term safety profile of *C. vulgaris* supplementation uncertain. Third, our experiments were conducted in healthy rats without tumor induction, so the possible interaction between *C. vulgaris* and cisplatin’s anti-tumor efficacy, especially in cancer models, remains to be clarified. Additionally, the complex composition of *C. vulgaris* means that the specific active components responsible for its protective effects were not isolated or characterized. Finally, comprehensive safety assessments, such as routine blood tests, urinalysis, and detailed histopathology of multiple organ systems, were not included. Future studies should address these limitations to provide a more complete understanding of both the safety and therapeutic potential of *C. vulgaris* supplementation.

## 5. Conclusions

In conclusion, *C. vulgaris* supplementation demonstrated substantial defenses counter to cisplatin-induced toxicity in rats, likely through its antioxidant, anti-inflammatory, and protective characteristics. While this study offers valuable insights into the protective effects of *C. vulgaris* supplementation against cisplatin-induced toxicity in experimental animals, further research is essential to fully understand its long-term safety and therapeutic potential. While these results are encouraging, further research including molecular mechanistic studies, long-term safety evaluations, and experiments in tumor-bearing models is necessary to fully establish the therapeutic potential and safety profile of *C. vulgaris* supplementation. These limitations should be considered when interpreting our findings.

## Figures and Tables

**Table 1:** Total phenolic and flavonoid compound of *C. vulgaris* supplement.

Retention Time (RT)	Compound	Concentration mg/g
13.1	gallic acid	10.6
27.8	catechin	5.6
31.49	chlorogenic acid	6.48
33.65	caffeic acid	32.78
48.8	hesperidin	6.00
50.37	rutin	12.83
52.46	ellagic acid	0.55

## Data Availability

The data presented in this study are available in the article.

## Supplementary Materials

The following supporting information can be downloaded at: https://www.mdpi.com/article/10.3390/life15060934/s1, Figure S1: HPLC chromatogram of *C. vulgaris* supplement.
