# Supplementary figures and images for: Protective Impacts of Chlorella vulgaris on Cisplatin-Induced Toxicity in Liver, Kidney, and Spleen of Rats: Role of Oxidative Stress, Inflammation, and Nrf2 Modulation
Source: Life (Basel). 2025 Jun 10;15(6):934. doi: 10.3390/life15060934 (PMC12194350; doi:10.3390/life15060934)

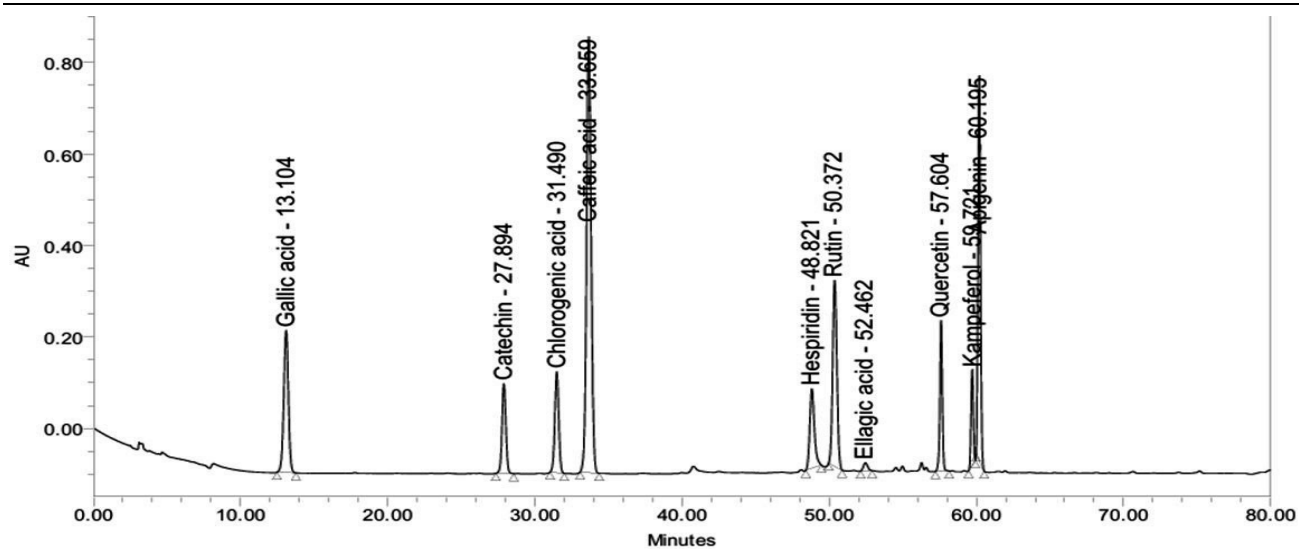

Figure S1. HPLC chromatogram of *C. vulgaris* supplement.

Supplement: Supplementary file 1 [file life-15-00934-s001.zip › life-3664692-suplementary.pdf]
